# LARP1-mediated stabilization of CD73 mRNA promotes immune escape in triple-negative breast cancer

**DOI:** 10.3389/fimmu.2026.1968375

**Published:** 2026-10-22

**Authors:** Xia Qiu, Jiying Shen, Yingxin Guan, Liping Chen, Jiahao Wu, Siyuan Li, Yibao Du, Yifan Gu

**Affiliations:** 1 Department of Breast Surgery, Tongren Hospital, Shanghai Jiao Tong University School of Medicine, Shanghai, China; 2 Department of General Surgery, Tongren Hospital, Shanghai Jiao Tong University School of Medicine, Shanghai, China

**Keywords:** CD73, gene-expression analysis, immune escape, LARP1, triple-negative breast cancer

## Abstract

**Background:**

Triple-negative breast cancer (TNBC) is characterized by limited therapeutic options and frequent immune escape, while the molecular mechanisms linking tumor-intrinsic programs with antitumor immunity remain incompletely understood.

**Methods:**

LARP1 was identified through integrative analysis of TNBC transcriptomic datasets and RNA-binding protein databases, and its broader clinical and prognostic relevance was evaluated in the METABRIC breast cancer cohort. Functional effects of LARP1 depletion or overexpression were evaluated in TNBC cells. Cell proliferation, apoptosis, CD73 expression, mRNA stability, and enrichment of NT5E mRNA in LARP1 immunoprecipitates were assessed. Activated human CD8+ T-cell co-culture, CD73 rescue experiments, and an immunocompetent 4T1 mouse model were used to evaluate immune regulation *in vitro* and *in vivo*.

**Results:**

LARP1 was elevated in breast cancer and TNBC-related expression comparisons. Exploratory analyses in the full TCGA-BRCA cohort suggested associations with survival and immune-cell composition, although LARP1 was not independently associated with overall survival after multivariable adjustment. In the independent METABRIC cohort, high LARP1 expression was associated with worse overall survival in both univariate and multivariable analyses (adjusted HR 1.422, 95% CI 1.215–1.664, P<0.001). LARP1 depletion suppressed TNBC-cell proliferation, promoted apoptosis, and enhanced CD8+ T-cell-mediated tumor-cell killing, whereas LARP1 overexpression increased proliferation and modestly reduced apoptosis. Mechanistically, LARP1 and NT5E expression showed a weak but statistically significant positive correlation in TCGA-BRCA, and LARP1 depletion reduced surface CD73 expression. CD73 mRNA was enriched in LARP1 immunoprecipitates and exhibited accelerated degradation following LARP1 loss, indicating that LARP1 maintains CD73 expression through mRNA stabilization. Restoration of CD73 attenuated the enhanced CD8+ T-cell activity induced by LARP1 depletion, including increased IFN-γ and granzyme B production and reduced PD-1 expression. *In vivo*, Larp1 silencing was associated with reduced 4T1 tumor growth, reduced Ki-67 and PCNA expression, increased CD8+ T-cell representation and proliferation within tumors, and decreased PD-1 expression on tumor-infiltrating CD8+ T cells.

**Conclusions:**

LARP1 promotes TNBC progression by sustaining NT5E/CD73 expression and modulating tumor–T-cell interactions. *In vivo*, LARP1 depletion was associated with reduced tumor growth and altered intratumoral CD8+ T-cell profiles. These findings support further investigation of the LARP1–CD73 axis in TNBC.

## Introduction

Triple-negative breast cancer (TNBC) lacks estrogen receptor, progesterone receptor and human epidermal growth factor receptor 2 expression and remains one of the most aggressive breast cancer subtypes (1, 2). Although chemotherapy and immune-checkpoint blockade have improved outcomes for selected patients, durable responses are limited by substantial intertumoral heterogeneity and the rapid establishment of an immunosuppressive tumor microenvironment (3–6). Tumor-cell-intrinsic programs can reduce antigen-dependent cytotoxicity, alter the abundance and state of tumor-infiltrating lymphocytes, and generate soluble metabolites that suppress effector T-cell activity (7–9). Identifying regulators that connect malignant-cell fitness with immune evasion may therefore reveal biomarkers and therapeutic vulnerabilities that are particularly relevant to TNBC.

CD73, encoded by NT5E, is a glycosylphosphatidylinositol-anchored ecto-5′-nucleotidase that converts extracellular AMP into adenosine (10, 11). Together with upstream ATP/ADP-hydrolyzing enzymes, CD73 establishes an adenosine-rich extracellular milieu that signals through adenosine receptors on immune cells. This signaling restrains T-cell receptor responses, cytokine secretion and cytolytic activity and can promote an exhausted phenotype (10–12). High CD73 activity has been linked to poor outcome, chemotherapy resistance and reduced antitumor immunity in breast cancer, including TNBC (13–15). CD73 is therefore not merely a surface marker: its abundance on malignant cells can directly determine the strength of a metabolically mediated immune checkpoint (16–19). However, the post-transcriptional mechanisms that sustain CD73 expression in TNBC remain incompletely defined.

RNA-binding proteins coordinate mRNA processing, localization, stability and translation and can rapidly reshape oncogenic signaling without changes in DNA sequence (20, 21). La-related protein 1 (LARP1) is an evolutionarily conserved RNA-binding protein containing La-module and DM15 RNA-interaction domains. LARP1 is best known for integrating nutrient-sensitive signaling with the fate and translation of selected transcripts, including 5′ terminal oligopyrimidine mRNAs, but it can also alter the stability of cancer-relevant transcripts through untranslated regions (22–25). Increased LARP1 expression has been associated with malignant growth, migration and apoptosis resistance in several solid tumors (26, 27). Whether LARP1 also controls immune-regulatory transcripts in breast cancer, and whether such regulation contributes to immune escape, has not been established. These observations led us to hypothesize that LARP1 may sustain CD73 expression by regulating the stability of its encoding transcript, NT5E, thereby linking post-transcriptional RNA regulation to impaired T-cell activity. This proposed relationship was tested in the present study.

In this study, we integrated independent transcriptomic cohorts and The Cancer Genome Atlas (TCGA) for candidate discovery and used the METABRIC cohort for independent clinical and prognostic validation of LARP1. Cell-line data and processed single-cell breast cancer data were further incorporated to characterize its biological and immunological relevance. Further screening of immune-regulatory molecules identified NT5E/CD73 as a potential downstream target associated with LARP1. We therefore investigated whether LARP1 regulates CD73 expression through modulation of NT5E mRNA stability. Human CD8+ T-cell co-culture and rescue experiments were used to determine whether CD73 mediates LARP1-dependent immune resistance. Finally, an immunocompetent 4T1/BALB/c model was used to assess tumor growth and intratumoral CD8+ T-cell states *in vivo*. The findings support a model in which LARP1 sustains CD73 expression by stabilizing NT5E mRNA, thereby coupling TNBC growth with suppression of CD8+ T-cell activity.

## Materials and methods

### Clinical cohort and molecular analyses

Gene-expression profiles and sample annotations were obtained from GSE38959, GSE53752 and TCGA Breast Invasive Carcinoma (TCGA-BRCA) (28, 29). The discovery comparisons comprised 30 TNBC and 13 normal breast samples in GSE38959, 51 TNBC/invasive ductal carcinoma and 25 normal samples in GSE53752, and 1,113 breast tumors and 113 adjacent normal tissues in TCGA-BRCA, as available in the downloaded matrices. Expression values were normalized within each cohort, and differential expression was evaluated using the corresponding microarray or RNA-sequencing framework. P values from differential-expression analyses were adjusted for multiple testing using the Benjamini–Hochberg false discovery rate (FDR) method. Genes with an FDR-adjusted P value <0.05 and an absolute log2 fold change >1 were intersected with a curated human RNA-binding protein list. GSE38959 and GSE53752 provided relatively large independent expression cohorts for differential-expression screening, but the deposited records did not contain the survival outcomes and sufficiently detailed clinicopathological annotations required for reliable univariate or multivariable prognostic modeling. TCGA-BRCA complemented these datasets by providing survival and clinicopathological data and was therefore used for candidate-gene expression, univariate and multivariable Cox regression, Kaplan–Meier overall survival (OS) and disease-specific survival (DSS), clinicopathological associations, and immune-cell deconvolution.

An independent Molecular Taxonomy of Breast Cancer International Consortium (METABRIC) cohort was additionally used to validate the clinical and prognostic relevance of LARP1 in a separate large breast cancer population. Gene-expression, overall survival and clinicopathological data were obtained from METABRIC. A total of 1,979 patients with available LARP1 expression and survival information were included. Patients were classified into low- and high-LARP1 expression groups. Clinicopathological characteristics were compared between the two groups, and the association between LARP1 expression and overall survival was evaluated using univariate and multivariable Cox proportional-hazards regression. Variables evaluated included age, tumor size, nodal status, pathological stage, PR status, ER status, HER2 status, histological grade, PAM50 subtype and LARP1 expression.

For TCGA-BRCA analyses, patients were divided according to median LARP1 expression. These immune-deconvolution analyses were exploratory and were used to describe associations in the full TCGA-BRCA cohort rather than to define a TNBC-specific immune phenotype. CIBERSORT-derived fractions for 22 immune-cell populations were compared between LARP1-high and LARP1-low tumors and correlated with LARP1 using Spearman analysis (30). Cell-line expression was surveyed using the Cancer Cell Line Encyclopedia (31). Protein-expression images were obtained from the Human Protein Atlas (32). For single-cell analyses, processed expression matrices, cell annotations and low-dimensional embeddings from Wu et al. were used solely to provide descriptive cell-type context for LARP1 and NT5E across annotated breast tumor cell populations; no new patient-level single-cell sequencing or independent cell-type-specific analysis was performed, and the dataset was not treated as independent mechanistic or prognostic validation (33).

### Human participants and ethical approval

Peripheral blood samples were obtained from healthy adult volunteers at Tongren Hospital, Shanghai Jiao Tong University School of Medicine after written informed consent. The use of human blood and associated experimental procedures was approved by the Ethics Committee of Tongren Hospital, Shanghai Jiao Tong University School of Medicine (approval no. 2026-391) and was conducted in accordance with the Declaration of Helsinki. Only de-identified samples were used. Peripheral blood mononuclear cells (PBMCs) were isolated by density-gradient centrifugation and processed immediately or cryopreserved under standardized conditions.

### Cell culture and lentiviral manipulation

Human TNBC MDA-MB-231 and BT-549 cells and murine 4T1 mammary carcinoma cells were maintained under supplier-recommended conditions at 37 °C in a humidified atmosphere containing 5% CO2. Cell identities were confirmed by short tandem repeat profiling where applicable, and cultures were routinely tested for mycoplasma. Lentiviral particles encoding human LARP1 short hairpin RNAs (shLARP1#1 and shLARP1#2), murine shLarp1, LARP1 overexpression, CD73 overexpression, or the corresponding controls were custom-produced by Tsingke Biotechnology (Beijing, China). Draft target sequences used for reporting were human shLARP1#1, 5′-GCGCCAGATTGAATACTACTT-3′; human shLARP1#2, 5′-CAAGAAGTCGAGAACTTCAAA-3′; murine shLarp1, 5′-TAGACATGAAGCCCGAAGTAC-3′; and non-targeting shNC, 5′-CAACAAGATGAAGAGCACCAA-3′. Cells were transduced in the presence of 8 μg/mL polybrene (Sigma-Aldrich, H9268) and selected with puromycin (Sigma-Aldrich, P8833). Knockdown or overexpression was verified before functional experiments.

### RNA extraction, quantitative reverse-transcription PCR, western blotting and RIP

Total RNA was isolated, reverse-transcribed and quantified by real-time PCR using SYBR chemistry. Relative expression was calculated by the 2−ΔΔCt method with GAPDH/Gapdh as the internal control. Primer pairs (5′–3′; synthesized by Sangon Biotech, Shanghai, China) were: human LARP1, forward TAGAGCGAGACTTCTTCCTGCG and reverse ATCTCCACCACCTTGCTGTCCT; human NT5E, forward AGTCCACTGGAGAGTTCCTGCA and reverse TGAGAGGGTCATAACTGGGCAC; human GAPDH, forward GGAGCGAGATCCCTCCAAAAT and reverse GGCTGTTGTCATACTTCTCATGG; mouse Larp1, forward CAAAAGTGTGCAGCCACAGTC and reverse CCCATTCTTTTCCTCCCCCG; mouse Nt5e, forward CAAATCTGCCTCTGGAAAGC and reverse ACCTTCCAGAAGGACCCTGT; and mouse Gapdh, forward AGGTCGGTGTGAACGGATTTG and reverse TGTAGACCATGTAGTTGAGGTCA. For western blotting, proteins were resolved by SDS-PAGE and transferred to PVDF membranes. After α-tubulin detection and imaging, the same membranes were treated with stripping buffer and then reprobed for LARP1 or CD73. Primary antibodies were LARP1 (Cell Signaling Technology [CST], 14763; 1:1,000), CD73 (CST, 13160; 1:1,000) and α-tubulin (Proteintech, 66031-1-Ig; 1:5,000), followed by HRP-conjugated anti-rabbit (CST, 7074) or anti-mouse (CST, 7076) secondary antibodies. RNA immunoprecipitation (RIP) was performed with the Magna RIP kit (Millipore, 17-700) using anti-LARP1 (Abcam, ab86359) or species-matched IgG. Co-precipitated NT5E mRNA was measured by qPCR and expressed relative to input and/or IgG. For mRNA-decay assays, transcription was blocked with actinomycin D (Sigma-Aldrich, A9415); RNA was collected at the indicated times, normalized to the baseline value, and fitted to a one-phase exponential-decay model to estimate NT5E mRNA half-life.

### Cell proliferation and apoptosis assays

For proliferation measurements, genetically modified cells were seeded at 2×10^3 cells per well in 96-well plates and analyzed at 0, 24, 48 and 72 hours using Cell Counting Kit-8 (Elabscience, E-CK-A362). Absorbance was measured at 450 nm after incubation with reagent according to the manufacturer’s instructions. Apoptosis was quantified by flow cytometry using Annexin V-FITC/propidium iodide staining (Elabscience, E-CK-A211). Cells positive for Annexin V with or without propidium iodide were considered apoptotic. Each cellular experiment was performed with at least three independent replicates.

### CD8+ T-cell isolation, activation, co-culture and flow cytometry

CD8+ T cells used in the co-culture experiments were isolated from PBMCs obtained from six independent healthy adult donors (n=6 donors). Human CD8+ T cells were purified from PBMCs using the CD8+ T Cell Isolation Kit (Miltenyi Biotec, 130-096-495), activated with Dynabeads Human T-Activator CD3/CD28 (Thermo Fisher Scientific, 11131D), and expanded in medium containing recombinant human interleukin-2 (PeproTech, 200-02). Activated effector cells were co-cultured with shNC, shLARP1 or shLARP1 plus CD73-overexpressing TNBC target cells at effector-to-target ratios of 1:1, 3:1 and 10:1 in this allogeneic co-culture system. Tumor-cell lysis was calculated relative to matched target-only controls after the assay interval. Surface CD73 on viable, non-permeabilized tumor cells was detected with FITC-conjugated anti-human CD73 (BioLegend, 344016; clone AD2) and reported as positive fraction and fluorescence distribution. T-cell analyses used APC-conjugated anti-human CD3 (BioLegend, 300312; clone HIT3a), PerCP/Cyanine5.5-conjugated anti-human CD8 (BioLegend, 344710; clone SK1), PE-conjugated anti-human IFN-γ (BioLegend, 506507; clone B27), FITC-conjugated anti-human/mouse granzyme B (BioLegend, 515403; clone GB11) and Pacific Blue-conjugated anti-human PD-1 (BioLegend, 329916; clone EH12.2H7). Intracellular cytokine or granule staining was performed after fixation and permeabilization using standard controls and compensation. Data were acquired by flow cytometry and analyzed using consistent gates across groups.

### Syngeneic tumor model, histology and tumor immune profiling

All animal procedures were approved by the Institutional Animal Care and Use Committee of Tongren Hospital, Shanghai Jiao Tong University School of Medicine (protocol no. A2026-132-01). Female BALB/c mice (6–8 weeks old) were randomized to receive 1×10^6 4T1-shNC or 4T1-shLarp1 cells by subcutaneous injection in the neck region (n=5 per group). No formal *a priori* power calculation was performed, and the animal experiment was designed as an exploratory *in vivo* assessment of tumor-growth and immune-profile changes following Larp1 depletion. Tumor dimensions were measured at the indicated intervals, and volume was calculated as length×width²/2. Mice were euthanized at the prespecified endpoint, and tumors were excised and weighed. Formalin-fixed paraffin-embedded sections were stained with hematoxylin and eosin and subjected to immunohistochemistry for Ki-67 (CST, 12202; clone D3B5) and proliferating cell nuclear antigen (PCNA; CST, 13110; clone D3H8P) using an HRP-based detection system (CST, 8114). For immune profiling, tumors were mechanically and enzymatically dissociated into single-cell suspensions. Murine leukocytes and T cells were identified with CD45 (BioLegend, 103114; 30-F11), CD3 (100204; 17A2) and CD8 (100752; 53-6.7); CD8+ T-cell proliferation and PD-1 expression were assessed with Ki-67 (652406; 16A8) and PD-1 (135206; 29F.1A12), respectively.

### Statistical analysis

Data are presented as mean ± SD unless stated otherwise. Two-group comparisons used two-tailed Student’s t tests or nonparametric alternatives when distributional assumptions were not met. Comparisons among three or more groups used one-way analysis of variance with appropriate multiple-comparison testing, and time-course experiments used two-way analysis of variance. For CCK-8 assays, time-course data were analyzed using two-way ANOVA. Between-group comparisons were performed at 72 h using Dunnett’s multiple-comparisons test, with multiplicity-adjusted P values reported. Categorical clinicopathological variables were evaluated using χ² or Fisher’s exact tests. Survival curves were compared by the log-rank test, and hazard ratios were estimated using Cox proportional-hazards models. Correlations were evaluated using Spearman coefficients. For analyses involving multiple genes or immune-cell populations, including the immune-regulatory gene comparisons shown in **Figure 1A**, P values were adjusted using the Benjamini–Hochberg FDR method, with FDR-adjusted P<0.05 considered statistically significant. All tests were two-sided.

**Figure 1:**
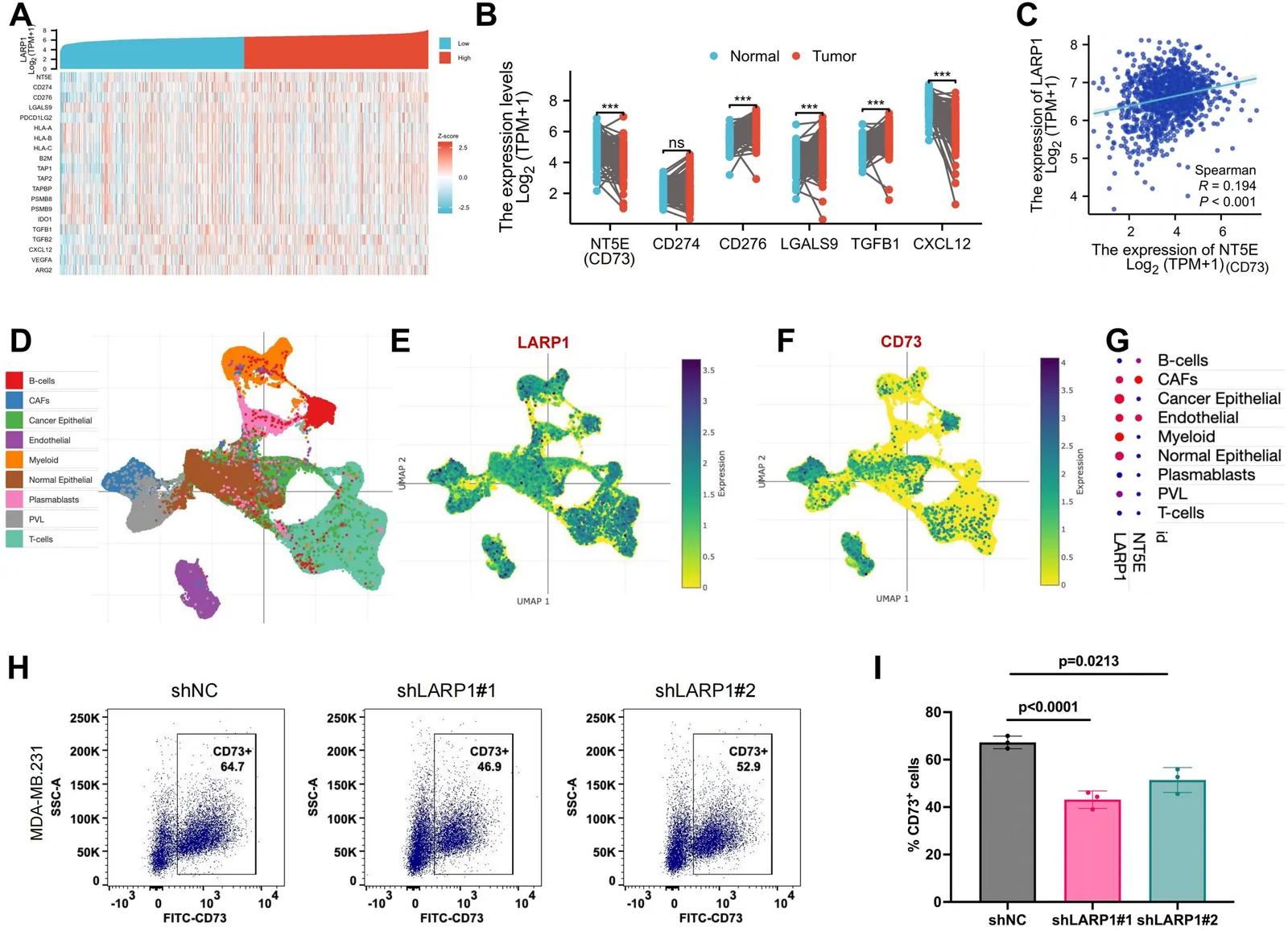
Integrated analyses identify CD73 as a LARP1-associated immune-regulatory molecule. **(A)** Heatmap of immune-regulatory genes in TCGA-BRCA tumors stratified by LARP1 expression. **(B)** Paired TCGA analysis of NT5E, CD274, CD276, LGALS9, TGFB1 and CXCL12 expression in breast tumor and adjacent tissue. **(C)** TCGA Spearman correlation between LARP1 and NT5E. **(D)** UMAP of annotated cell populations in the processed human breast cancer single-cell dataset from Wu et al. (Nature Genetics 2021;53:1334–1347; doi:10.1038/s41588-021-00911-1). **(E, F)** Single-cell feature plots for LARP1 and CD73/NT5E. **(G)** Dot plot summarizing LARP1 and NT5E expression across annotated cell types. **(H)** Representative flow-cytometric distributions of surface CD73 in MDA-MB-231 shNC, shLARP1#1 and shLARP1#2 cells. **(I)** Quantification of surface CD73-positive cells. Data in I are mean ± SD from three independent experiments. Statistical significance in panel **(A)** is based on Benjamini–Hochberg FDR-adjusted P values. CAF, cancer-associated fibroblast; PVL, perivascular-like cell; UMAP, uniform manifold approximation and projection.

## Results

### Integrated transcriptomic screening identifies LARP1 as a candidate RBP in breast cancer

To identify RNA-binding proteins associated with TNBC, we first compared tumor and normal breast transcriptomes in two independent Gene Expression Omnibus cohorts and TCGA-BRCA. Differential-expression analyses showed that LARP1 was differentially expressed in both GSE38959 and GSE53752, within the broader transcriptional changes observed across these cohorts and TCGA-BRCA (**Figures 2A–C**). Intersecting the three differential-gene sets with a curated RBP list produced 28 shared candidates, including LARP1 (**Figure 2D**). The GEO cohorts were used for independent differential-expression screening but lacked the survival outcomes and sufficiently detailed clinicopathological annotations needed for reliable prognostic modeling; therefore, survival and Cox regression analyses were performed in TCGA-BRCA. In TCGA univariate Cox analysis, five of these genes were associated with OS: LARP1, POP1, GGCT and EZR were adverse factors, whereas CIRBP was protective (**Figure 2E**; **Supplementary Table S1**). Among the candidates, LARP1 was consistently increased in TNBC-related comparisons and was selected because it combined differential expression, survival association and a plausible post-transcriptional regulatory function (**Figure 2F**). Human Protein Atlas images further supported higher LARP1 protein staining in breast ductal carcinoma than in normal breast tissue (**Figure 2G**).

**Figure 2:**
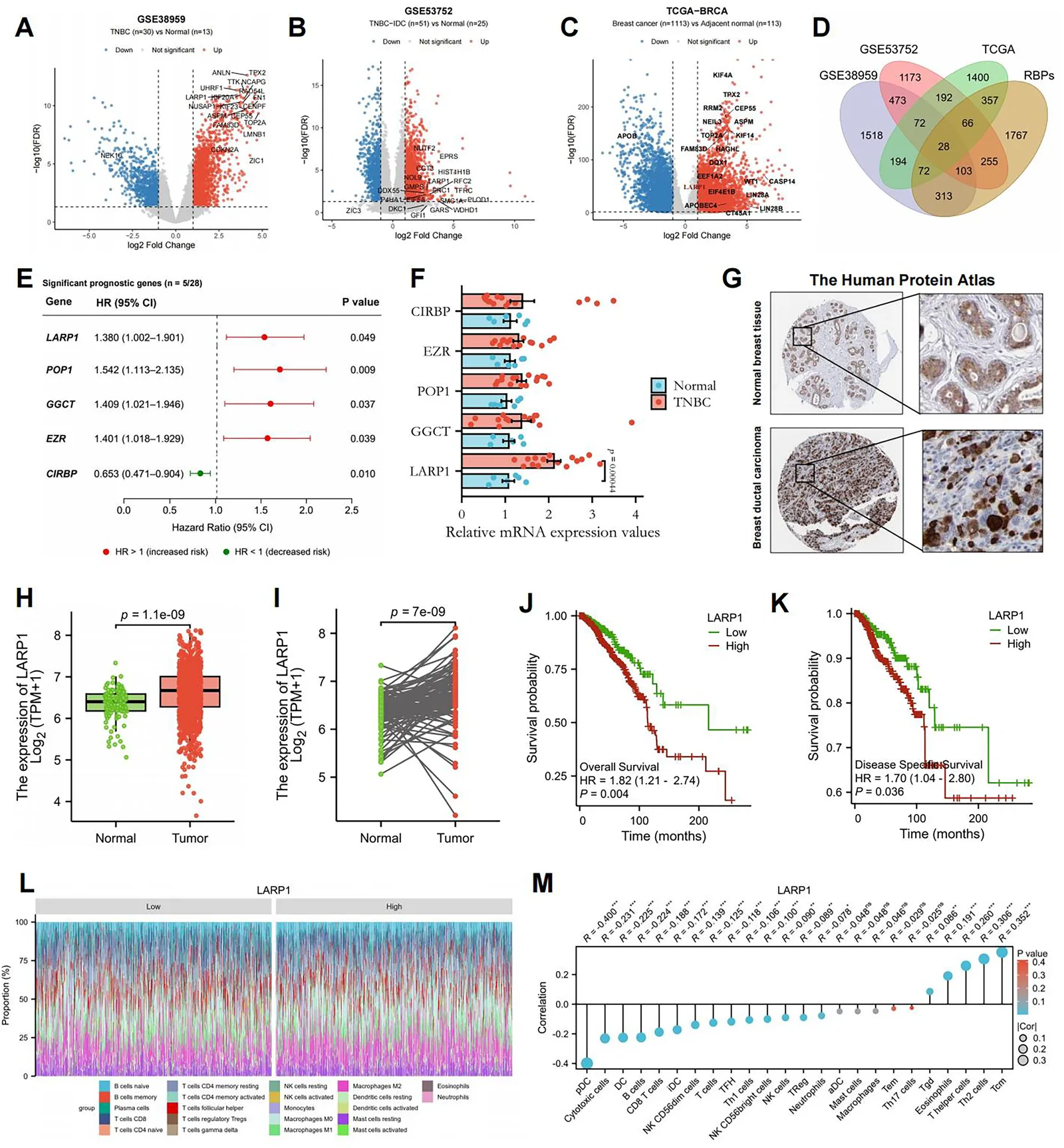
Integrated identification and clinical characterization of LARP1 in breast cancer. **(A)** Volcano plot comparing 30 TNBC and 13 normal breast samples in GSE38959. **(B)** Volcano plot comparing 51 TNBC/invasive ductal carcinoma and 25 normal samples in GSE53752. **(C)** Differential-expression analysis of 1,113 breast tumors and 113 adjacent normal tissues in TCGA-BRCA. **(D)** Intersection of differentially expressed genes from the three cohorts with a curated RNA-binding protein list identified 28 shared candidates. **(E)** TCGA univariate Cox analysis of the five candidates associated with overall survival. **(F)** TCGA expression comparison of the five survival-associated candidates in normal and TNBC samples. **(G)** Representative LARP1 immunohistochemistry images from the Human Protein Atlas. **(H, I)** TCGA LARP1 expression in unpaired and paired breast tumor and normal tissues. **(J, K)** Kaplan–Meier overall survival and disease-specific survival according to TCGA LARP1 expression. **(L)** CIBERSORT-estimated immune-cell composition in LARP1-low and LARP1-high TCGA tumors. **(M)** Spearman correlations between LARP1 and inferred immune-cell fractions in TCGA. Differential-expression and immune-cell correlation P values were adjusted for multiple testing using the Benjamini–Hochberg FDR method, where applicable. BRCA, breast invasive carcinoma; DSS, disease-specific survival; OS, overall survival; RBP, RNA-binding protein; TCGA, The Cancer Genome Atlas; TNBC, triple-negative breast cancer.

### LARP1 expression is associated with outcome across independent cohorts and an altered immune contexture

TCGA analyses confirmed higher LARP1 RNA expression in unpaired breast tumors and in matched tumor–normal pairs (**Figures 2H, I**). Patients with high LARP1 expression had shorter OS and DSS in unadjusted Kaplan–Meier analyses (**Figures 2J, K**). In the full TCGA-BRCA clinicopathological dataset, LARP1 category was associated with estrogen- and progesterone-receptor status but not with T, N or M category or overall pathological stage, underscoring the influence of molecular-subtype composition in a pan-breast-cancer cohort (**Table 1**). LARP1 showed a borderline association with OS in univariate analysis (HR 1.380, P = 0.049), whereas this association was not significant after multivariable adjustment (HR 0.917, P = 0.72).

**Table 1:** Clinicopathological characteristics of the TCGA-BRCA cohort according to LARP1 expression level.

Characteristics	Available cases (n)	Low LARP1 expression	High LARP1 expression	P value
Age, n (%)	1087			0.378
≤60 years		294 (54.1%)	309 (56.8%)	
>60 years		249 (45.9%)	235 (43.2%)	
Pathologic T stage, n (%)	1084			0.271
T1-T2		462 (42.6%)	447 (41.2%)	
T3–T4		81 (7.5%)	94 (8.7%)	
Pathologic N stage, n (%)	1065			0.158
N0		270 (50.5%)	246 (46.1%)	
N1–N3		265 (49.5%)	287 (53.9%)	
Pathologic M stage, n (%)	925			0.533
M0		434 (97.5%)	471 (98.1%)	
M1		11 (2.5%)	9 (1.9%)	
Pathologic stage, n (%)	1063			0.875
Stage I–II		405 (75.4%)	396 (75.1%)	
Stage III–IV		131 (24.6%)	131 (24.9%)	
PR status, n (%)	1039			<0.001
Negative		207 (39.8%)	135 (26.3%)	
Positive		313 (60.2%)	379 (73.7%)	
ER status, n (%)	1037			<0.001
Negative		154 (29.5%)	86 (16.7%)	
Positive		368 (70.5%)	429 (83.3%)	
HER2 status, n (%)	717			0.979
Negative		274 (78.1%)	286 (78.1%)	
Positive		77 (21.9%)	80 (21.9%)	

Percentages were calculated based on patients with available clinical information for each variable. Missing data were excluded from the analysis.

To further evaluate the clinical relevance of LARP1 in an independent cohort, we next analyzed the METABRIC cohort. A total of 1,979 patients with available LARP1 expression and survival data were included. The LARP1-high and LARP1-low groups differed significantly with respect to age, tumor size, PR status, HER2 status, histological grade and PAM50 subtype, whereas nodal status, pathological stage and ER status did not differ significantly between the groups (**Table 2**). In univariate Cox regression, high LARP1 expression was associated with worse overall survival (HR 1.570, 95% CI 1.385–1.781, P<0.001). Importantly, this association remained significant after multivariable adjustment for clinicopathological factors (HR 1.422, 95% CI 1.215–1.664, P<0.001; **Table 3**). These findings provide independent cohort-level support for the association between elevated LARP1 expression and adverse outcome in breast cancer.

**Table 2:** Clinicopathological characteristics of the METABRIC cohort according to LARP1 expression level.

Characteristics	Available cases (n)	Low LARP1 expression	High LARP1 expression	P value
Age, n (%)	1979			0.003
≤60 years		359 (48.4%)	513 (41.5%)	
>60 years		383 (51.6%)	724 (58.5%)	
Tumor size, n (%)	1954			<0.001
≤2 cm		361 (49.2%)	491 (40.2%)	
>2 cm		373 (50.8%)	729 (59.8%)	
Nodal status, n (%)	1903			0.160
N0		387 (54.3%)	605 (50.8%)	
N1–N3		326 (45.7%)	585 (49.2%)	
Pathologic stage, n (%)	1454			0.956
Stage I–II		509 (91.1%)	817 (91.3%)	
Stage III–IV		50 (8.9%)	78 (8.7%)	
PR status, n (%)	1979			0.010
Negative		324 (43.7%)	615 (49.7%)	
Positive		418 (56.3%)	622 (50.3%)	
ER status, n (%)	1979			0.162
Negative		164 (22.1%)	309 (25.0%)	
Positive		578 (77.9%)	928 (75.0%)	
HER2 status, n (%)	1979			0.001
Negative		673 (90.7%)	1059 (85.6%)	
Positive		69 (9.3%)	178 (14.4%)	
Histologic grade, n (%)	1892			<0.001
Grade 1–2		423 (59.3%)	517 (43.9%)	
Grade 3		290 (40.7%)	662 (56.1%)	
PAM50 subtype, n (%)	1973			<0.001
Non-Luminal A		439 (59.3%)	834 (67.6%)	
Luminal A		301 (40.7%)	399 (32.4%)	

P values were calculated using the χ² test.

**Table 3:** Univariate and multivariate Cox regression analyses of prognostic factors associated with overall survival in the METABRIC cohort.

Characteristics	Available cases (n)	HR (95% CI), univariate	P value	HR (95% CI), multivariate	P value
Age (>60 vs ≤60)	1979	1.941 (1.713–2.199)	<0.001	1.994 (1.703–2.334)	<0.001
Tumor size (>2 cm vs ≤2 cm)	1954	1.749 (1.548–1.975)	<0.001	1.463 (1.250–1.712)	<0.001
Nodal status (N1–N3 vs N0)	1903	1.669 (1.482–1.880)	<0.001	1.463 (1.253–1.707)	<0.001
Stage (III–IV vs I–II)	1454	2.363 (1.908–2.927)	<0.001	1.512 (1.192–1.917)	<0.001
PR (Positive vs Negative)	1979	0.792 (0.705–0.890)	<0.001	0.940 (0.791–1.117)	0.481
ER (Positive vs Negative)	1979	0.862 (0.751–0.989)	0.035	0.871 (0.703–1.079)	0.205
HER2 (Positive vs Negative)	1979	1.467 (1.238–1.738)	<0.001	1.410 (1.131–1.758)	0.002
Grade (3 vs 1–2)	1892	1.337 (1.186–1.506)	<0.001	1.000 (0.846–1.182)	0.999
PAM50 (Luminal A vs Non-Luminal A)	1973	0.741 (0.655–0.839)	<0.001	0.903 (0.758–1.076)	0.254
LARP1 (High vs Low)	1979	1.570 (1.385–1.781)	<0.001	1.422 (1.215–1.664)	<0.001

HR, hazard ratio; CI, confidence interval. Cox proportional hazards regression analyses were performed to evaluate factors associated with overall survival. .

Immune deconvolution revealed different leukocyte compositions in LARP1-high and LARP1-low tumors (**Figure 2L**). LARP1 correlated negatively with plasmacytoid dendritic cells and showed negative relationships with several cytotoxic, dendritic-cell, B-cell and CD8+ T-cell signals, while positive correlations were observed with selected T-helper and other immune populations (**Figure 2M**; **Supplementary Table S2**). These exploratory findings describe immune associations in the full TCGA-BRCA cohort and provide broader immune-context information complementary to the TNBC-focused experimental findings. The hazard ratio shown in **Figure 2J** derives from the unadjusted high-versus-low survival comparison, whereas **Table 4** reports the Cox estimate based on complete cases available for the modeled variables.

**Table 4:** Univariate and multivariate Cox regression analyses of prognostic factors associated with overall survival in the TCGA-BRCA cohort.

Characteristics	Available cases (n)	HR (95% CI), univariate	P value	HR (95% CI), multivariate	P value
Age (≤60 years vs >60 years)	1086	2.024 (1.468–2.790)	<0.001	3.065 (1.850–5.077)	<0.001
Pathologic T stage (T1 vs T2–T4)	1083	1.480 (1.005–2.179)	0.047	1.024 (0.575–1.825)	0.935
Pathologic N stage (N0 vs N1–N3)	1067	2.232 (1.563–3.189)	<0.001	1.298 (0.704–2.396)	0.404
Pathologic M stage (M0 vs M1)	925	4.266 (2.474–7.354)	<0.001	2.675 (1.020–7.016)	0.045
Pathologic stage (Stage I–II vs Stage III–IV)	1062	2.367 (1.686–3.321)	<0.001	2.979 (1.554–5.711)	0.001
PR status (Negative vs Positive)	1033	0.729 (0.521–1.019)	0.065	0.880 (0.408–1.896)	0.743
ER status (Negative vs Positive)	1036	0.709 (0.493–1.019)	0.063	0.518 (0.232–1.154)	0.107
HER2 status (Negative vs Positive)	717	1.593 (0.973–2.609)	0.064	0.935 (0.520–1.681)	0.823
LARP1 expression (Low vs High)	1086	1.380 (1.002–1.901)	0.049	0.917 (0.569–1.477)	0.72

HR, hazard ratio; CI, confidence interval. Cox proportional hazards regression analyses were performed to evaluate factors associated with overall survival. The first category of each variable was used as the reference group.

### LARP1 depletion suppresses TNBC-cell fitness and increases susceptibility to CD8+ T-cell killing

CCLE data were used to select MDA-MB-231 and BT-549 as complementary human TNBC models with detectable LARP1 expression (**Figure 3A**). Two independent shRNAs reduced LARP1 expression by more than 70% in both cell lines, with knockdown efficiency shown in **Figures 3B, C**. LARP1 depletion slowed cell accumulation over 72 hours and increased Annexin V-positive fractions, demonstrating that endogenous LARP1 supports TNBC-cell proliferation and survival (**Figures 3D–F**). We next isolated and activated CD8+ T cells from healthy-donor PBMCs and co-cultured them with control or LARP1-depleted targets at graded effector-to-target ratios (**Figure 3G**). Tumor-cell lysis increased with the effector-to-target ratio in both cell lines. At non-saturating ratios, shLARP1 targets were more efficiently lysed than shNC targets, whereas the difference narrowed at 10:1 as cytotoxicity approached a plateau (**Figure 3H**). Thus, LARP1 depletion reduces tumor-cell fitness and is associated with increased susceptibility to activated human CD8+ T-cell-mediated killing.

**Figure 3:**
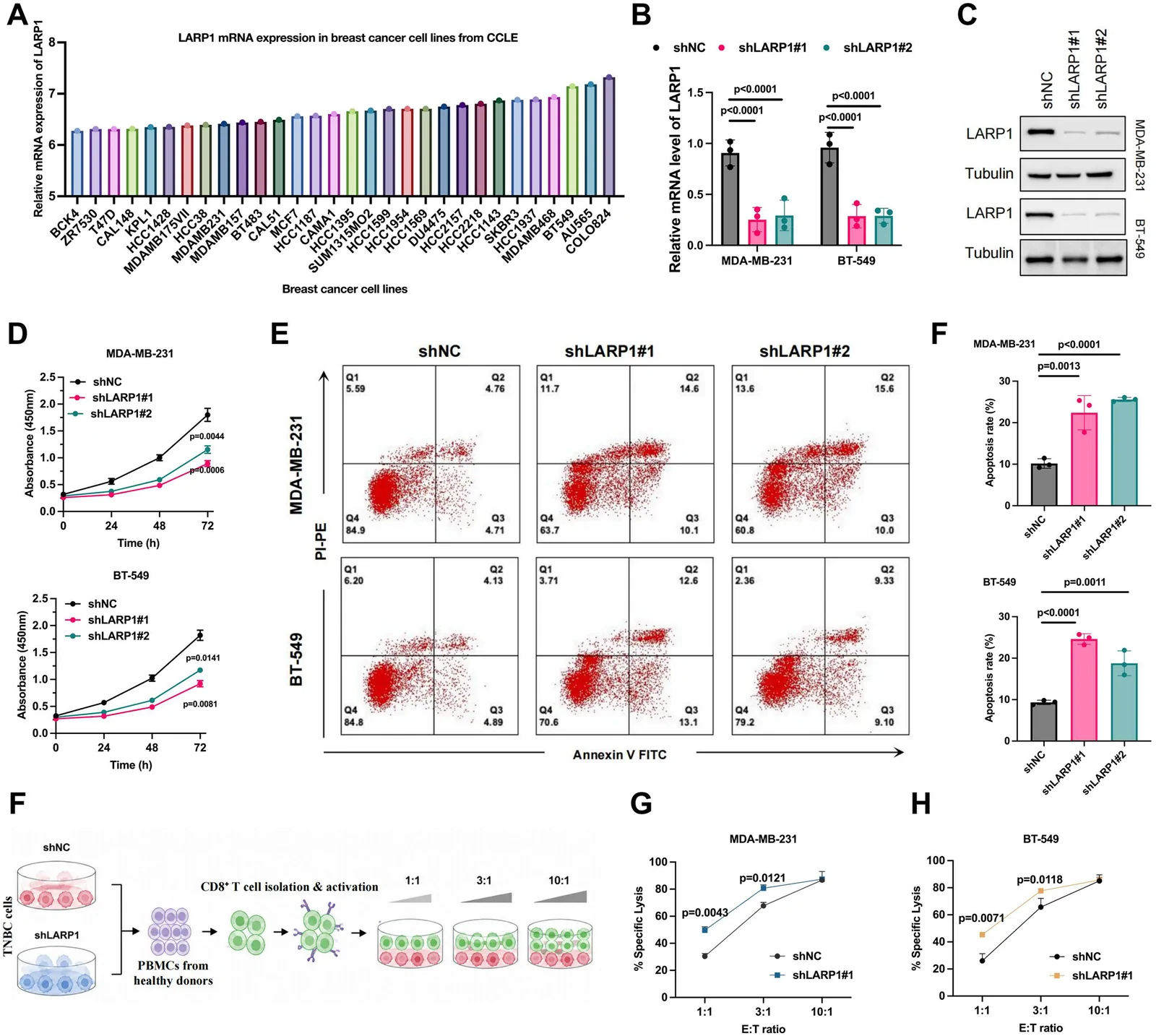
LARP1 depletion suppresses TNBC-cell fitness and enhances CD8+ T-cell-mediated killing. **(A)** CCLE survey of LARP1 mRNA expression across breast cancer cell lines. **(B, C)** LARP1 knockdown efficiency in MDA-MB-231 and BT-549 cells assessed by qPCR and western blotting. **(D)** CCK-8 proliferation curves after transduction with shNC, shLARP1#1 or shLARP1#2. **(E)** Representative Annexin V-FITC/propidium iodide flow plots. **(F)** Quantification of apoptotic cells. **(G)** Schematic of healthy-donor PBMC isolation, CD8+ T-cell activation and co-culture with genetically modified TNBC cells at effector-to-target ratios of 1:1, 3:1 and 10:1. **(H)** Tumor-cell lysis of MDA-MB-231 and BT-549 target cells. Data are mean ± SD from three independent experiments; P values are shown in the image. CCLE, Cancer Cell Line Encyclopedia; E:T, effector-to-target ratio; PBMC, peripheral blood mononuclear cell; PI, propidium iodide.

### LARP1 overexpression reinforces malignant phenotypes in TNBC cells

To complement the loss-of-function experiments, LARP1 was ectopically expressed in MDA-MB-231 and BT-549 cells. Increased LARP1 transcript and protein abundance was confirmed by qPCR and western blotting (**Figures 4A, B**). LARP1 overexpression accelerated cell accumulation in both models, with the largest separation from control cultures at 72 hours (**Figures 4C, D**). In parallel, the proportion of Annexin V-positive cells showed a small decrease following LARP1 overexpression (**Figures 4E, F**). The knockdown and overexpression results support a role for LARP1 in TNBC-cell proliferation, with a modest effect of overexpression on apoptosis.

**Figure 4:**
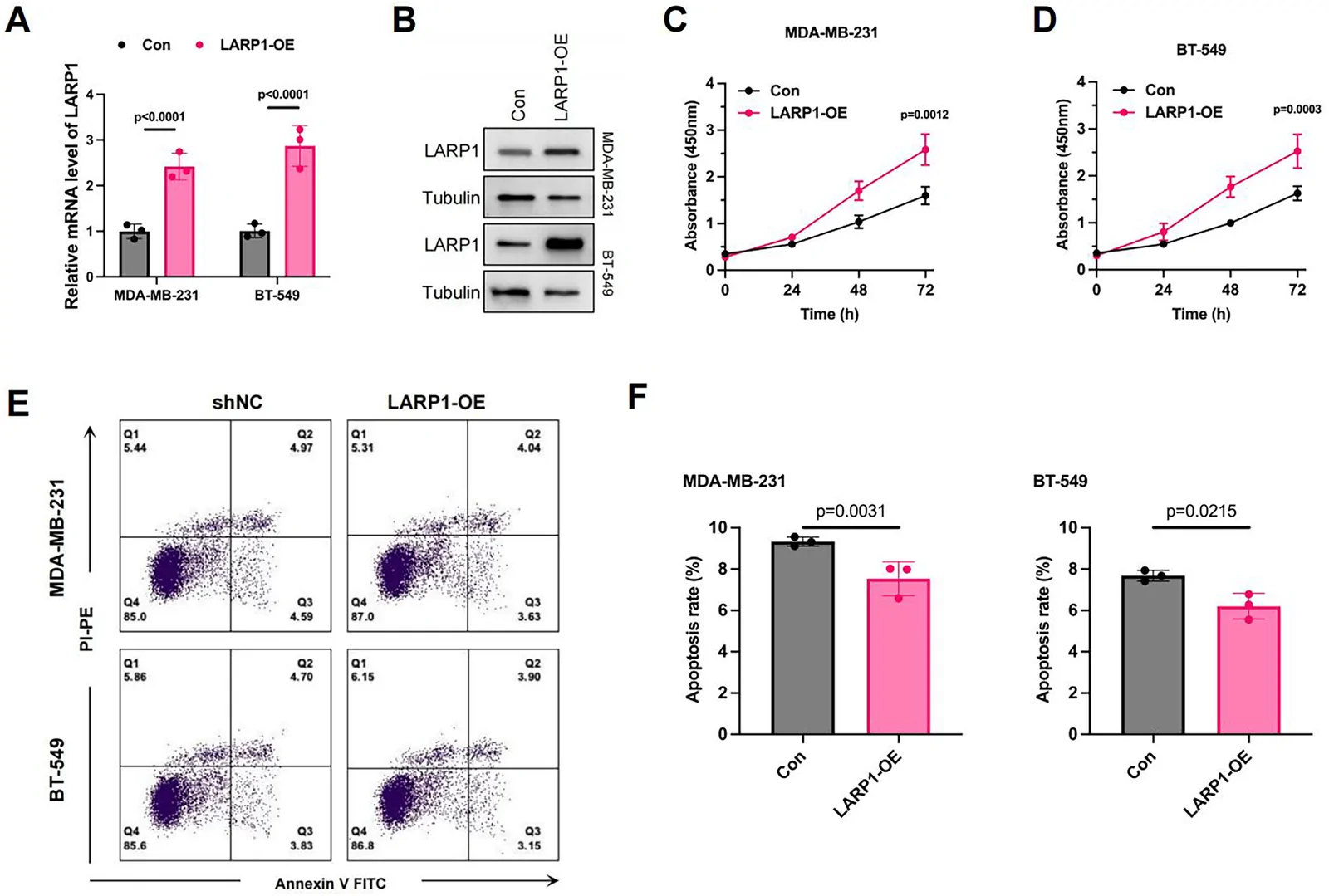
LARP1 overexpression promotes TNBC-cell proliferation and survival. **(A, B)** Confirmation of LARP1 overexpression in MDA-MB-231 and BT-549 cells by qPCR and western blotting. **(C, D)** CCK-8 proliferation curves for control and LARP1-overexpressing cells. **(E)** Representative Annexin V-FITC/propidium iodide apoptosis plots. **(F)** Quantification of apoptotic fractions in the two cell lines. Data are mean ± SD from three independent experiments; P values are shown in the image.

### Multi-omic analyses nominate CD73 as an immune-regulatory target of LARP1

We next investigated how LARP1 might connect tumor-cell behavior with immune suppression. In TCGA-BRCA, LARP1-high and LARP1-low tumors showed coordinated differences across immune-regulatory genes, including NT5E, CD274, CD276, LGALS9, TGFB1 and CXCL12 (**Figure 1A**). Paired TCGA comparisons confirmed that several of these candidates were differentially expressed between tumor and adjacent tissue; importantly, the directions were gene-specific rather than uniformly increased in tumors (**Figure 1B**). LARP1 and NT5E expression showed a modest but highly significant positive correlation (Spearman r=0.194, P<0.001; **Figure 1C**). Analysis of the processed single-cell breast cancer atlas from Wu and colleagues mapped epithelial, stromal and immune compartments and demonstrated heterogeneous LARP1 and CD73 expression across the tumor ecosystem, with overlapping expression in malignant and selected stromal populations (**Figures 1D–G**). To assess whether LARP1 depletion alters cell-surface CD73 abundance, we directly measured non-permeabilized TNBC cells. LARP1 knockdown reduced the surface CD73-positive fraction and shifted CD73 fluorescence downward in MDA-MB-231 cells, with concordant effects from both shRNAs (**Figures 1H, I**).

### LARP1 stabilizes NT5E mRNA and CD73 restoration rescues LARP1-loss phenotypes

We then tested whether LARP1 controls NT5E transcript stability. After actinomycin D-mediated transcriptional arrest, NT5E mRNA decayed more rapidly in LARP1-depleted cells than in controls. In MDA-MB-231 cells, the estimated half-life fell from 4.235 hours in shNC cells to 1.921 and 2.689 hours with shLARP1#1 and shLARP1#2, respectively; in BT-549 cells, the corresponding values were 3.601, 2.544 and 1.690 hours (**Figures 5A, B**). Thus, although the magnitude of the effect differed between the two shRNAs and cell lines, both shRNAs consistently shortened NT5E mRNA half-life relative to shNC in each model. RIP-qPCR showed marked enrichment of NT5E mRNA in anti-LARP1 immunoprecipitates relative to IgG in both cell lines, placing NT5E transcripts within LARP1-containing ribonucleoprotein complexes (**Figure 5C**). To test the functional relevance of CD73 downstream of LARP1 depletion, CD73 was re-expressed in LARP1-depleted MDA-MB-231 cells. CD73 restoration rescued the proliferation defect caused by LARP1 loss (**Figure 5D**). In CD8+ T-cell co-cultures, LARP1 depletion increased IFN-γ-positive and granzyme B-positive CD8+ T cells and reduced PD-1-positive CD8+ T cells; restoring CD73 reversed each phenotype toward the shNC state (**Figures 5E–G**). To further exclude a hairpin-specific effect, the CD73 rescue experiment was independently repeated in BT-549 cells using shLARP1#2. CD73 re-expression restored cell proliferation and reversed the shLARP1#2-associated increases in IFN-γ- and granzyme B-positive CD8+ T cells and the reduction in PD-1-positive CD8+ T cells (**Figures 5H–K**). These findings reproduced the rescue phenotype obtained with shLARP1#1 and provide additional support for a specific LARP1-CD73 regulatory relationship. These data identify CD73 as a functional mediator linking LARP1-dependent NT5E mRNA stability to impaired CD8+ T-cell activity.

**Figure 5:**
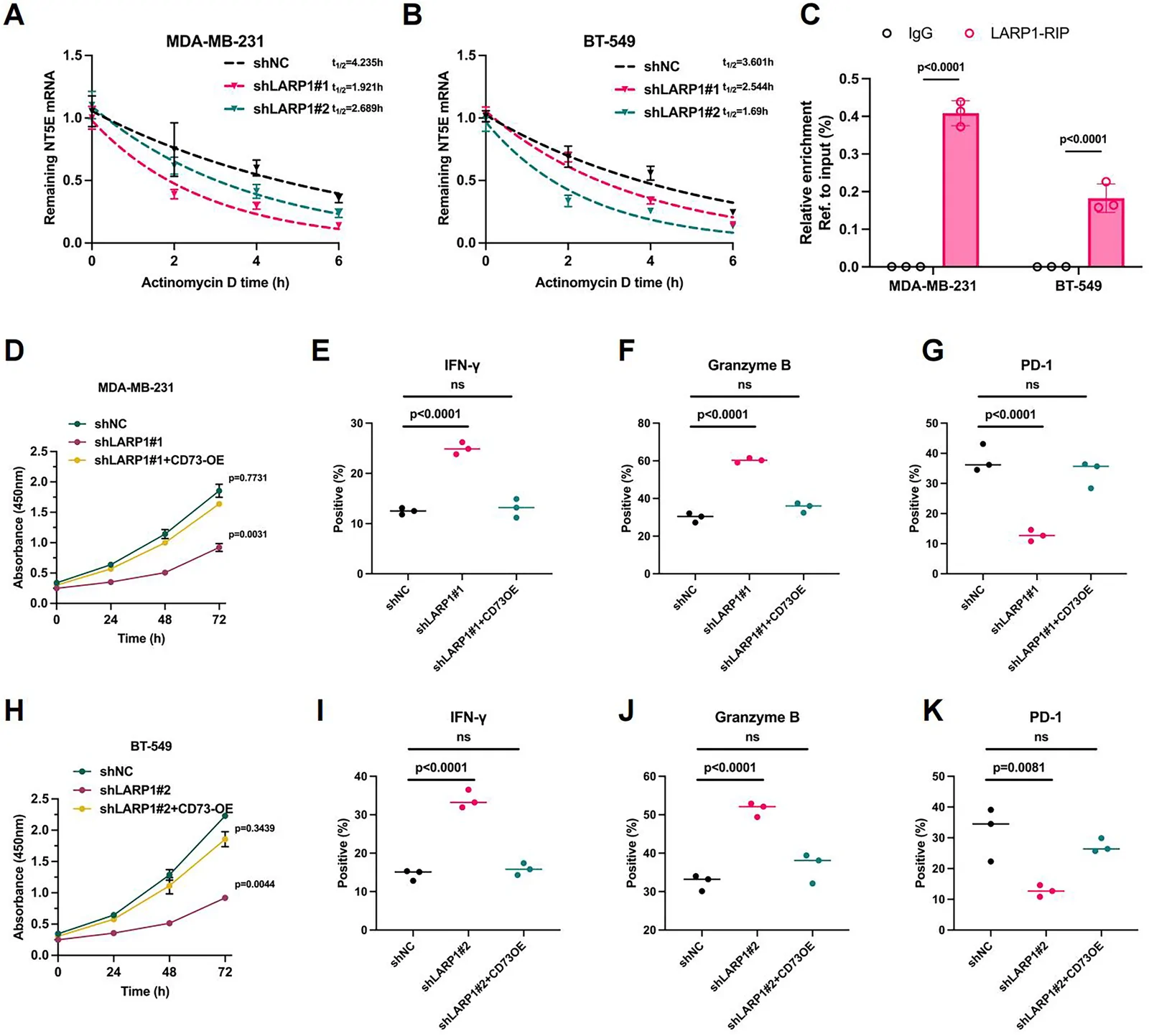
LARP1 stabilizes NT5E mRNA and CD73 mediates LARP1-dependent tumor and immune phenotypes. **(A, B)** Actinomycin D chase assays showing normalized NT5E mRNA decay and estimated half-lives in MDA-MB-231 and BT-549 shNC, shLARP1#1 and shLARP1#2 cells. **(C)** RIP-qPCR detection of NT5E mRNA in IgG and anti-LARP1 immunoprecipitates from both TNBC cell lines. **(D)** CCK-8 proliferation assay in MDA-MB-231 shNC, shLARP1#1 and shLARP1#1 cells with CD73 re-expression. **(E–G)** Frequencies of IFN-γ-positive, granzyme B-positive and PD-1-positive CD8+ T cells after co-culture with the indicated tumor cells. CD73 re-expression reversed the T-cell phenotypes induced by LARP1 depletion. **(H–K)** Independent validation of the CD73 rescue using shLARP1#2 in BT-549 cells. Data are mean ± SD from three independent experiments; P values are shown. ActD, actinomycin D; GZMB, granzyme B; RIP, RNA immunoprecipitation.

### Larp1 depletion restricts 4T1 tumor growth and alters intratumoral CD8+ T-cell profiles

The LARP1 phenotype was finally evaluated in an immunocompetent syngeneic setting. BALB/c mice received neck-subcutaneous injections of 4T1-shNC or 4T1-shLarp1 cells (**Figure 6A**). Larp1-depleted tumors grew more slowly throughout the 35-day observation period and had lower terminal wet weights than control tumors (**Figures 6B, C**). Histological analysis showed reduced Ki-67 and PCNA staining after Larp1 depletion, consistent with diminished proliferation *in vivo* (**Figures 6D–F**). Flow cytometric profiling demonstrated a higher proportion of CD8+ cells among intratumoral CD3+ T cells and an increased Ki-67-positive fraction within the CD8+ compartment in shLarp1 tumors (**Figures 6G, H**). Conversely, PD-1 expression on intratumoral CD8+ T cells was reduced, as illustrated by representative distributions and group quantification (**Figures 6I, J**). Together, the *in vivo* data show that Larp1 loss is associated with reduced tumor growth, increased CD8+ T-cell representation and proliferation, and lower PD-1 expression on intratumoral CD8+ T cells, and lower PD-1 expression on CD8+ T cells. These *in vivo* observations are descriptive and do not establish that tumor control depends on CD73 or CD8+ T cells, or that T-cell exhaustion is reduced.

**Figure 6:**
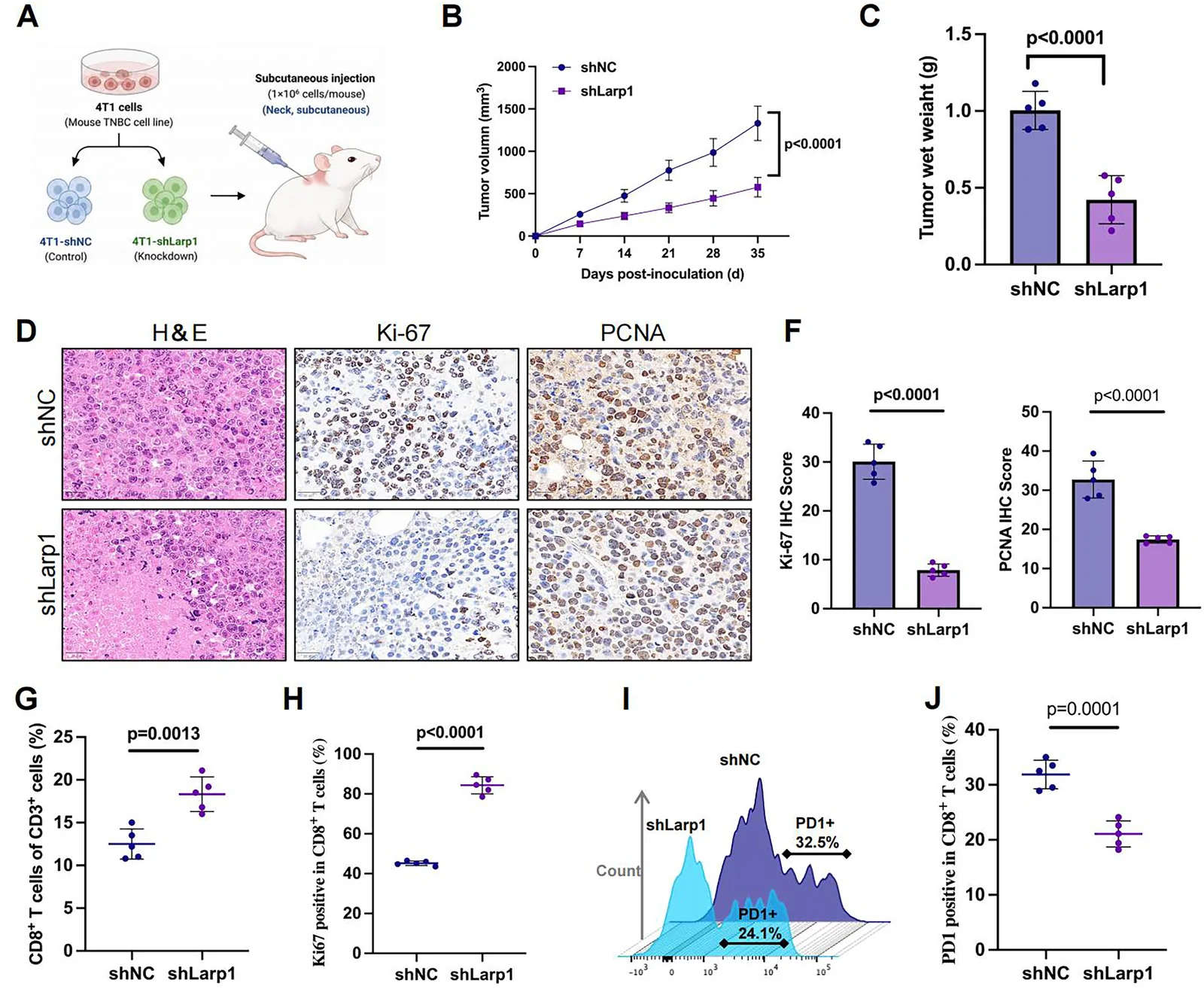
Larp1 depletion inhibits 4T1 tumor growth and alters intratumoral CD8+ T-cell profiles. **(A)** Schematic of the syngeneic model: BALB/c mice received neck-subcutaneous injections of 1×10^6 4T1-shNC or 4T1-shLarp1 cells. **(B)** Tumor growth curves through day 35. **(C)** Terminal tumor wet weights. **(D)** Representative hematoxylin and eosin, Ki-67 and PCNA staining of harvested tumors; scale bar, 20 μm. **(E, F)** Quantification of Ki-67 and PCNA immunohistochemical scores. **(G)** Flow-cytometric quantification of CD8+ T cells among intratumoral CD3+ T cells. **(H)** Ki-67-positive cells among intratumoral CD8+ T cells. **(I)** Representative PD-1 distributions in intratumoral CD8+ T cells. **(J)** Quantification of PD-1-positive CD8+ T cells. n=5 mice per group; data are mean ± SD; P values are shown. H&E, hematoxylin and eosin; PCNA, proliferating cell nuclear antigen.

## Discussion

This study identifies LARP1 as a post-transcriptional regulator linking TNBC-cell fitness to CD73-associated tumor–immune interactions. The conclusion is supported by a layered design: reproducible discovery across transcriptomic cohorts and independent prognostic validation in METABRIC; bidirectional perturbation of LARP1 in two TNBC cell lines; direct measurement of surface CD73; NT5E mRNA-decay and RIP assays; CD73 rescue in tumor–T-cell co-culture; and assessment of tumor growth and CD8+ T-cell profiles in an immunocompetent model (**Figure 7**). Taken together, these data extend the biological role of LARP1 beyond a generic growth-promoting RBP and support its role as an upstream regulator of CD73 expression and associated immune phenotypes *in vitro*.

**Figure 7:**
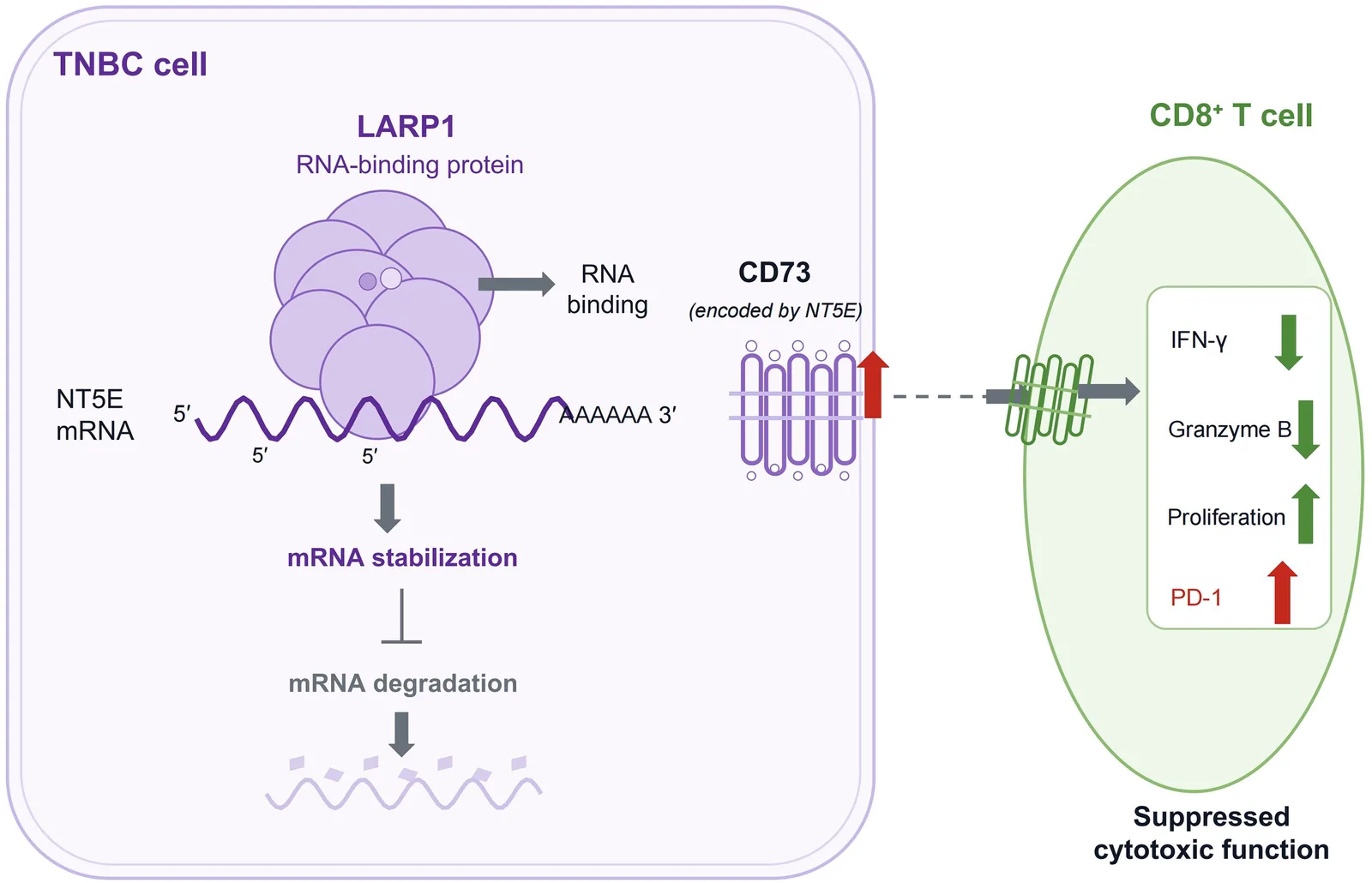
LARP1 stabilizes NT5E mRNA to maintain CD73 expression and suppress CD8+ T-cell cytotoxic function in TNBC. LARP1, an RNA-binding protein upregulated in TNBC, is associated with NT5E mRNA-containing ribonucleoprotein complexes and contributes to NT5E mRNA stability, leading to increased CD73 expression. Elevated CD73 expression is associated with impaired CD8+ T-cell cytotoxic activity, characterized by reduced IFN-γ and granzyme B production and increased PD-1 expression.

Previous work has implicated LARP1 in the control of mRNA translation and stability downstream of nutrient-sensitive signaling (20, 21, 23). Its DM15 region can recognize structured RNA features and cap-proximal motifs, whereas other studies have shown that LARP1 can stabilize or destabilize cancer-related transcripts through untranslated regions (22, 24, 25). In ovarian and other cancer models, LARP1-dependent regulation of survival-associated mRNAs has been linked to apoptosis resistance and aggressive behavior (26, 27). Our loss- and gain-of-function findings in MDA-MB-231 and BT-549 cells are consistent with this broader oncogenic pattern. The present work adds NT5E to the LARP1-associated transcript network and shows that the consequence is not limited to cell-autonomous proliferation: it also changes how tumor cells interact with cytotoxic lymphocytes.

The NT5E half-life measurements provide a mechanistic bridge between LARP1 abundance and surface CD73. In both TNBC lines, transcriptional blockade exposed faster NT5E decay after LARP1 depletion, while RIP-qPCR enriched NT5E mRNA in LARP1 immunoprecipitates. These complementary observations support a model in which LARP1-containing ribonucleoprotein complexes protect NT5E transcripts from degradation, thereby sustaining CD73 production. The differences in effect magnitude between the two shRNAs and cell lines may reflect variation in knockdown efficiency or cellular context and should not be overinterpreted. Importantly, RIP demonstrates association within a native complex but does not by itself prove direct contact or identify a binding motif. Because CLIP-seq, PAR-CLIP, or eCLIP was not performed in the present study, the specific NT5E region potentially involved in LARP1-associated regulation remains undefined. Crosslinking-based mapping, reporter assays containing the NT5E 5′UTR or 3′UTR, mutational analyses, and RNA pull-down experiments will be required to determine whether specific NT5E regions are sufficient to confer LARP1-dependent transcript stability and whether additional cofactors participate (34).

Our findings also align with the established immunobiology of CD73. Tumor-cell CD73 converts AMP into adenosine, which can suppress CD8+ T-cell receptor signaling, cytokine production and cytolytic granule release through adenosine receptors (10–12). In TNBC, high CD73 has previously been associated with poor prognosis, chemotherapy resistance and weak antitumor immunity, and pharmacological or genetic interference with this pathway can enhance immune responses in preclinical models (13–15, 34). The current rescue experiments provide causal evidence at the level of the proposed axis: LARP1 depletion increased IFN-γ and granzyme B and reduced PD-1 in CD8+ T cells, whereas re-expression of CD73 reversed those changes. The simultaneous rescue of tumor-cell proliferation suggests that CD73 may support both immune-mediated resistance and tumor-cell-intrinsic fitness, a dual function that could magnify its importance in LARP1-high tumors (17, 18, 35). The current rescue experiments establish CD73 as a functional mediator of LARP1-dependent immune regulation. However, they do not distinguish whether the observed effects are specifically mediated by the catalytic activity of CD73 and extracellular adenosine production or by other CD73-associated mechanisms. Further studies using catalytically inactive CD73 will be valuable to resolve this mechanistic distinction. In addition, healthy-donor T cells and established tumor cell lines were not HLA-matched; alloreactivity may contribute to the observed killing, which should not be interpreted as tumor-antigen-specific cytotoxicity. Nevertheless, the reversal of these co-culture phenotypes by CD73 re-expression supports a modulatory role of the LARP1–CD73 axis within this experimental system.

In TCGA-BRCA, LARP1 was associated with adverse survival in univariate analysis but did not retain statistical significance after multivariable adjustment. Importantly, analysis of the independent METABRIC cohort provided additional validation: high LARP1 expression was associated with worse overall survival in univariate analysis and remained significant after adjustment for clinicopathological factors. The differences in effect estimates between TCGA-BRCA and METABRIC may reflect differences in cohort composition, expression platforms, follow-up characteristics and available clinicopathological covariates. Collectively, the independent METABRIC analysis reduces the likelihood that the observed survival association is specific to a single dataset. Nevertheless, because these survival analyses were performed in broad breast cancer cohorts rather than TNBC-restricted populations, they do not establish LARP1 as an independent TNBC-specific prognostic biomarker.

Likewise, the LARP1–NT5E correlation was significant but modest. These findings are expected for a regulator operating within a heterogeneous bulk-tumor ecosystem and caution against treating LARP1 expression as a stand-alone clinical biomarker. Hormone-receptor associations in the pan-breast-cancer cohort also reflect subtype composition and should not be extrapolated directly to TNBC. The single-cell atlas provided cellular context, without resolving subtype confounding, by showing that both genes are distributed across malignant and stromal compartments (33). Future spatial or cell-type-specific analyses will be needed to determine whether the clinically relevant interaction is strongest in cancer epithelial cells or is reinforced by CD73-expressing stromal populations. More broadly, recent studies have identified distinct malignant-cell-associated programs that couple tumor fitness with suppression of antitumor immune responses, further illustrating the diversity of tumor-associated immune-evasion mechanisms (36).

The 4T1 experiments extend the observations to an intact immune system. Larp1 depletion reduced tumor volume and weight, decreased Ki-67 and PCNA, increased the relative abundance and proliferation of intratumoral CD8+ T cells, and reduced PD-1 expression. These findings suggest that the *in vivo* antitumor effect of Larp1 depletion may reflect combined tumor-cell-intrinsic and immune-associated mechanisms, although their relative contributions cannot be distinguished in the current model. These patterns agree with the human co-culture findings and support an immune-relevant phenotype associated with Larp1 depletion *in vivo*. Notably, the increased representation and proliferation of intratumoral CD8+ T cells together with reduced PD-1 expression are biologically consistent with the established immunosuppressive role of CD73. Previous studies have demonstrated that tumor-cell CD73 suppresses antitumor T-cell responses and that targeting CD73 can enhance adaptive and CD8+ T-cell-dependent antitumor immunity, including in breast cancer and 4T1-derived models (12–14, 37). These findings provide biological context for the immune phenotype observed in our model, while the relative contribution of CD73- and CD8+ T-cell-mediated mechanisms to the reduction in tumor growth remains to be further defined. Testing the axis in orthotopic mammary-fat-pad models and additional syngeneic TNBC systems would also strengthen generalizability. The neck-subcutaneous implantation site does not reproduce the mammary tissue microenvironment and limits extrapolation to orthotopic breast tumors. PD-1 alone cannot distinguish exhaustion from recent T-cell activation; additional markers such as TOX, TIM-3 or LAG-3 and functional assessments are needed to characterize exhaustion. Complementary LARP1-overexpression CD8+ T-cell co-culture experiments were performed in parallel; however, the observed trend toward reduced CD8+ T-cell-mediated cytotoxicity did not reach statistical significance under the current experimental conditions. Therefore, the immune co-culture findings are interpreted primarily on the basis of the LARP1-depletion experiments.

From a translational perspective, the LARP1–CD73 axis offers two conceptually different intervention points. Targeting CD73 or adenosine signaling acts at the extracellular effector layer and may be combined with PD-1/PD-L1 blockade, whereas interfering with LARP1 could reduce both malignant-cell fitness and the supply of CD73 transcripts (16, 19, 35, 38). The latter strategy may have a broader therapeutic window challenge because LARP1 also participates in normal RNA and translational homeostasis. Biomarker-guided approaches will therefore be essential. Tumors with high LARP1, preserved surface CD73 and a low-cytotoxicity immune profile may be the most rational candidates for testing CD73-directed combinations (10, 11, 38). Before clinical translation, the predictive value of the axis should be evaluated in independent TNBC cohorts and patient-derived models and against measured responses to immunotherapy (3–5, 7). Recent work further highlights the importance of treatment context and tumor–immune dynamics when considering immune-modulatory therapeutic strategies (39).

## Conclusion

In conclusion, the *in vitro* data support a model in which LARP1 associates with NT5E mRNA-containing complexes, stabilizes the transcript and sustains tumor-cell surface CD73. LARP1 depletion reduces tumor-cell growth and enhances CD8+ T-cell effector activity, while CD73 restoration reverses key *in vitro* phenotypes. In the 4T1 model, Larp1 depletion was associated with reduced tumor growth and an altered intratumoral CD8+ T-cell profile characterized by increased representation and proliferation and lower PD-1 expression. Together with the *in vitro* mechanistic findings, these observations support further investigation of the LARP1–CD73 axis in TNBC. Together with the *in vitro* mechanistic findings, these observations support further investigation of the LARP1–CD73 axis in TNBC.

## Data Availability

The original contributions presented in the study are included in the article/**Supplementary Material**. Further inquiries can be directed to the corresponding author.
